# Dataset for atmospheric transport of nutrients during a harmful algal bloom

**DOI:** 10.1016/j.dib.2020.105839

**Published:** 2020-06-07

**Authors:** Rongxiang Tian, Qun Lin, Dewang Li, Wei Zhang, Xiuyi Zhao

**Affiliations:** a School of Earth Sciences, Zhejiang University, Hangzhou 310027, China; b Key Laboratory of Marine Ecosystem and Biogeochemistry, Second Institute of Oceanography, State Oceanic Administration, Hangzhou 310012, China; c College of Software Technology, Zhejiang University, Ningbo 315103, China

**Keywords:** Harmful algal blooms, Atmospheric transport, Nutrients, The East China Sea

## Abstract

The data presented in this article are related to the research article entitled “Atmospheric transport of nutrient matter during a harmful algal bloom”[1]. These data provide the concentration of nutrients (nitrate, ammonium and Fe^Ⅱ^) in the atmosphere and their deposited flux in the East China Sea prior to the harmful algal bloom on May 3–8, 2006. They can be helpful for analyzing the source of nutrients causing the harmful algal blooms.

Specifications table

**Table:** 

Subject	Environmental Science (General)
Specific subject area	harmful algal bloom, Atmospheric transport, nutrient matter, numerical simulation, deposition flux, the East China Sea.
Type of data	Figure
How data was acquired	Survey and computer simulation using the Global Nested Air Quality Prediction Modeling System
Data format	Raw and Analyzed
Description of data collection	• The large-scale harmful algal bloom events were collected from the Marine Environmental Quality Bulletin of China (http://www.coi.gov.cn/hygb) • Global Nested Air Quality Prediction Modeling System [2], [3], [4] were used to simulate the atmospheric transport and deposition flux of nutrient.
Parameters for data collection	• The harmful algal bloom event with an area larger than 1000km^2^ in the East China Sea during 2006. • The harmful algal bloom event (a spatial coverage of 1000km^2^), which developed in coast water off Zhejiang, China between the 3rd- the 8th of May 2006, was use as research case. Then the Global Nested Air Quality Prediction Modeling System was used to simulate the atmospheric transport and deposition flux of nutrient in algal bloom area.
Data source location	Zhejiang Province, China
Data accessibility	The data is available within this article. https://data.mendeley.com/datasets/mh54hnp52f/draft?a=d6217d12-3d9d-45d6–8050–43f55a5d2e8e
Related research article	Rongxiang Tian, Qun Lin, Dewang Li, Wei Zhang, Xiuyu Zhao. Atmospheric transport of nutrients during a harmful algal bloom event[J]. Regional Studies in Marine Science, 2019: 101,007, DOI: https://doi.org/10.1016/j.rsma.2019.101007

## Value of the Data

• The data could be used to assess the contribution of the atmospheric nutrients to algal blooms
• The data can be used to identify areas of elevated nutrient concentrations and deposition flux in the East China Sea.
• The data can be used as a reference for predicting harmful algal bloom.

## Data description

**Fig. 1:**
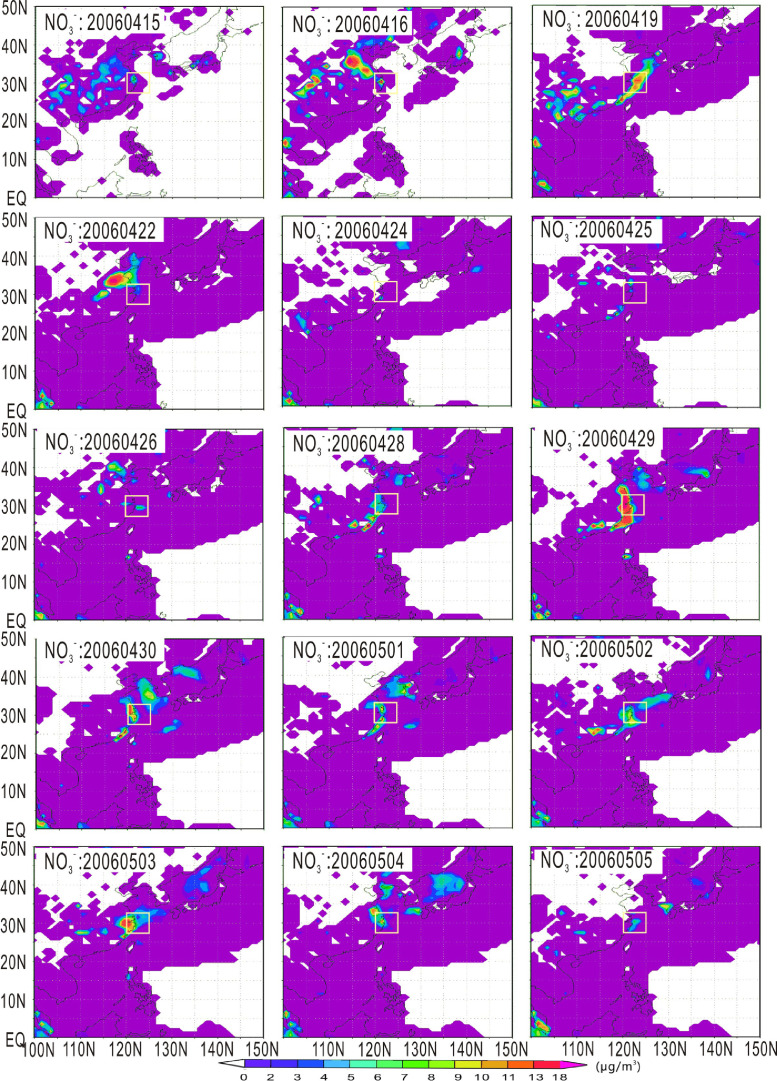
Atmospheric transport of nitrate (μg/m^3^). The small square represents the location of the 28–32°N, 120–124°E.

**Fig. 2:**
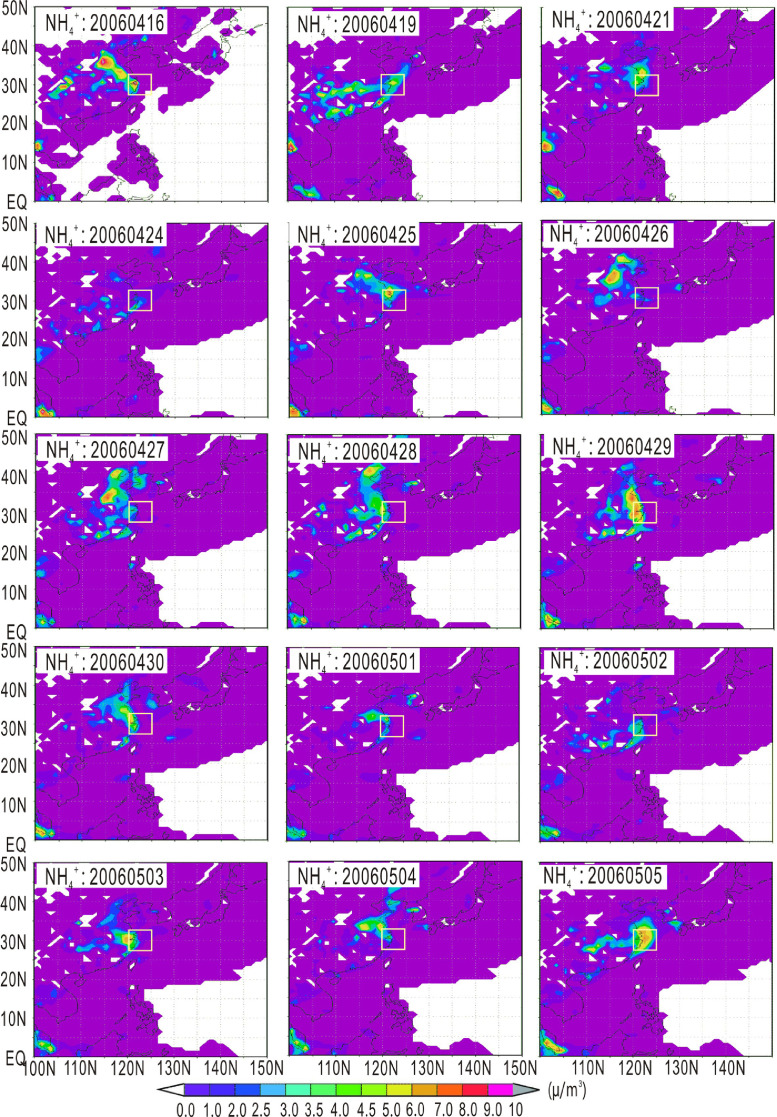
Atmospheric transport of ammonium (μg/m^3^). The small square represents the location of the 28–32°N, 120–124°E.

**Fig. 3:**
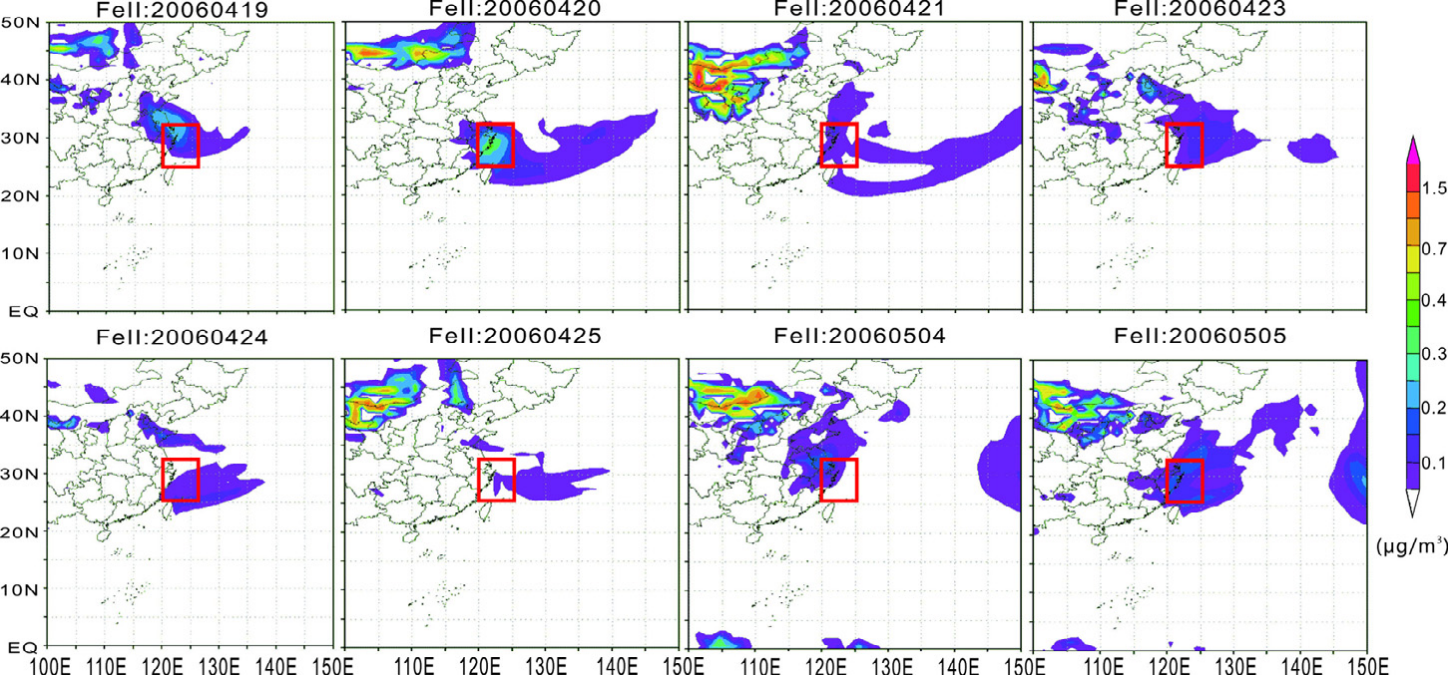
Atmospheric transport of Fe^Ⅱ^(μg/m^3^) [1]. The small square represents the location of the 28–32°N, 120–124°E.

**Fig. 4:**
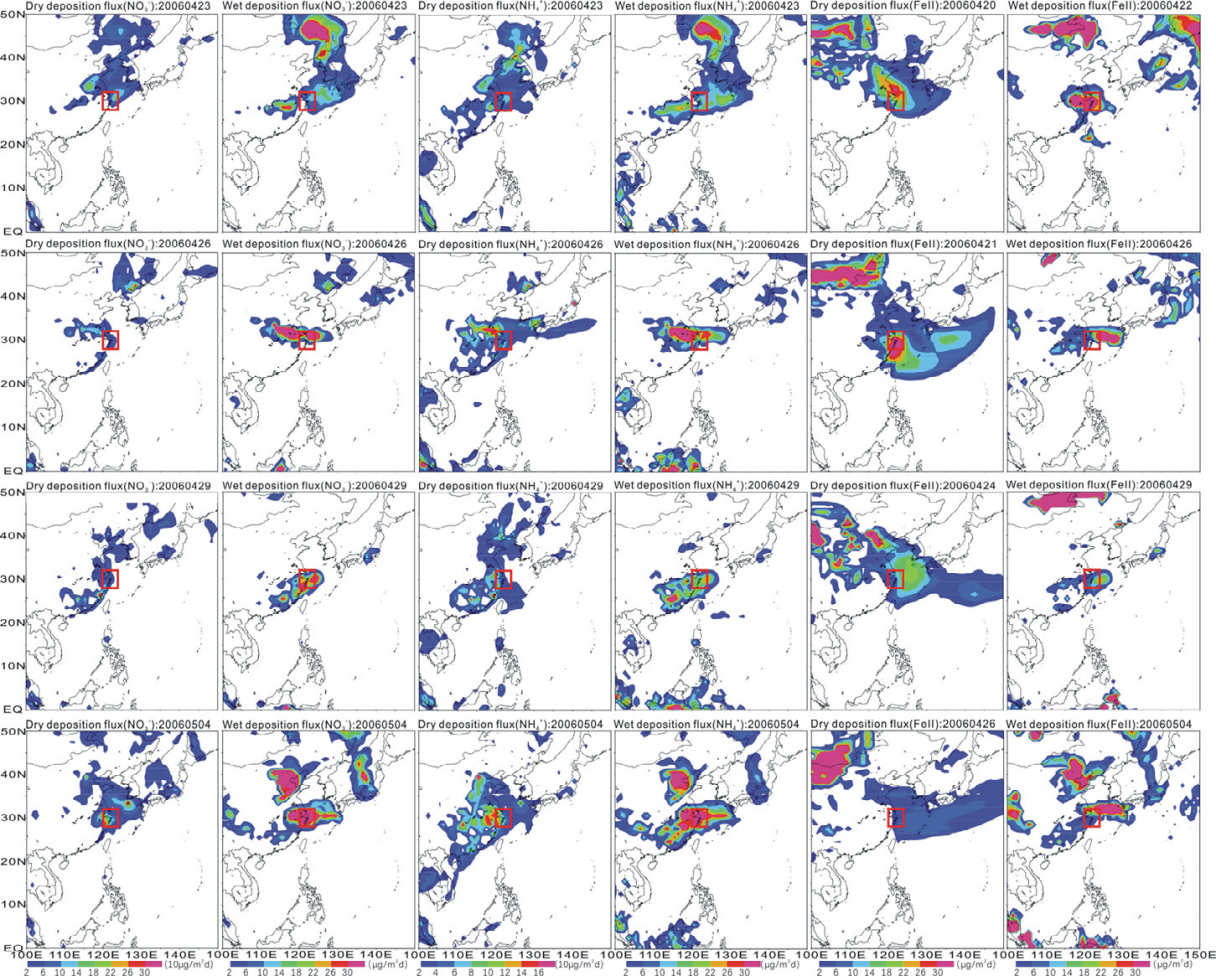
Deposition flux of nutrients. The small square represents the location of the 28–32°N, 120–124°E.

The harmful algal bloom event developed between May 3 and 8, 2006 was the first bloom with a spatial coverage of 1000 km^2^ that year. The considerable nutrient matter (nitrate, ammonium, ferrous iron) were transported to the algal bloom area via the atmosphere before occurring the harmful algal bloom. Fig. 1, Fig. 2, Fig. 3 show the Atmospheric transport of nitrate, ammonium and Fe^Ⅱ^, respectively. Fig. 4 shows the deposition flux of nutrients.

## Experimental design, materials and methods

There were 6 harmful algal bloom events which were larger than 1000 km^2^ in East China Sea during 2006. The first harmful algal bloom with a spatial coverage of 1000 km^2^ developed on May 3–8, 2006. April and May are the transition time between winter and summer in the Northern Hemisphere. The atmospheric circulation patterns and oceanic currents in East China Sea were shifting from winter patterns to summer patterns during this period. In consideration of the seasonal change and time when the first harmful algla bloom with a spatial coverage of 1000km^2^, we used the Global Nested Air Quality Prediction Modeling System (GNAQPMS) to calculate the atmospherically transported nutrients (nitrate, ammonium and Fe^Ⅱ^) and their deposition flux by setting the simulation time from April 15 to May 6, 2006.

The GNAQPMS was independently developed by Institute of Atmospheric Physics, Chinese Academy of Sciences (IAP/CAS)[5] . The GNAQPMS model and its running schedule were provided by IAP/CAS [2]. A summary is presented here:

The GNAQPMS utilized in this study is a fully modularized three-dimensional regional Eulerian chemical transport model, driven by the meteorological model the Weather Research and Forecasting (WRF) model. GNAQPMS reproduces the physical and chemical evolution of reactive pollutants by solving the mass balance equation in terrain-following coordinates [5, [7], [8], [9]]. It includes advection, diffusion and convection processes, gas/aqueous/aerosol chemistry, and parameterization of dry/wet deposition. GNAQPMS is composed of input, physic-chemical process and output:

**Input.** The input items are the meteorological fields and emission inputs. The output of the WRF model was used for the meteorological fields. The meteorological data on April 15, 2006, as the initial meteorological fields inputted to the WRF model, comes from http://rda.ucar.edu. The emission inputs consist of anthropogenic inputs (aerosol and trace gas) and natural emissions (vegetation, soil, volcano and lighting). In this study, we used the Fifth Assessment Report of the United Nations Intergovernmental Panel on Climate Change as anthropogenic input (1850–2000 decade, 0.5° × 0.5°), Global Emissions Inventory Activity and Model of Emissions of Gases and Aerosols from Nature as biological input (2000 decade, 0.5° × 0.5°). Regional Emission inventory in Asia as soil NOx input (year of 2001, 1° × 1°) and Global Emissions Inventory Activity as lighting NOx input (average value from 1983–1990, 1° × 1°)[6].

**Physic-chemical process.** The physical-chemical process included gas/aqueous/ heterogeneous/aerosol chemistry, advection, diffusion and convection processes, and modules for dry and wet deposition, and dust and sea salt dynamic emissions reactions and processes [4, 6, 9].

**Output.** The output items included the wet and dry deposition and spatial distribution of chemical species [6].

The GNAQPMS had been modified on the basis of the topography and pollution pattern of East Asia. It has been widely applied to simulate the transport of air pollutants and to provide operational air quality forecasts in East Asia [3, 4, [7], [8], [9], [10], [11], [12], [13], [14], [15]]

## Declaration of Competing Interest

The authors declare that we have no known competing financial interests or personal relationships that could have appeared to influence the work reported in this paper.
